# Oncological Properties of Intravenous Leiomyomatosis: Involvement of Mesenchymal Tumor Stem-Like Cells

**DOI:** 10.3390/cimb43020084

**Published:** 2021-09-19

**Authors:** Saya Tamura, Takuma Hayashi, Hideki Tokunaga, Nobuo Yaegashi, Kaoru Abiko, Ikuo Konishi

**Affiliations:** 1 National Hospital Organization Kyoto Medical Center, Department of Obstetrics and Gynecology, Kyoto 612-8555, Japan; nho.kmc.rc@hotmail.com (S.T.); hiroyukiaburatani@yahoo.co.jp (K.A.); ikuokonishi08@yahoo.co.jp (I.K.); 2 Section of Cancer Medicine, National Hospital Organization Kyoto Medical Center, Kyoto 612-8555, Japan; 3 START-Program, Japan Science and Technology Agency (JST), Tokyo 102-8666, Japan; 4 Department of Obstetrics and Gynecology, Tohoku University School of Medicine, Miyagi 980-8575, Japan; kenjisano12@yahoo.co.jp (H.T.); nobuoyaegashi@yahoo.co.jp (N.Y.); 5 Department of Obstetrics and Gynecology, Kyoto University School of Medicine, Kyoto 606-8501, Japan

**Keywords:** intravenous leiomyomatosis, leiomyoma, leiomyosarcoma, tumor stem-like cells

## Abstract

Uterine leiomyoma, also known as fibroids, is the most common benign neoplasm of the female genital tract. Leiomyoma is the most common uterine tumor. The leiomyoma subtypes account for approximately 10% of leiomyomas. Intravenous leiomyomatosis, a uterine leiomyoma subtype, is an intravascular growth of benign smooth muscle cells, occasionally with pelvic or extrapelvic extension. Uterine leiomyosarcoma, a malignant tumor, tends to metastasize hematogenously, and distant metastasis to the lungs and liver is common. Therefore, the oncological properties of this intravenous leiomyomatosis resemble those of the malignant tumor uterine leiomyosarcoma. Cancer stem cells migrate to distant organs via intravascular infiltration, leading to micrometastases. We examined the oncological properties of intravenous leiomyomatosis using molecular pathological techniques on tissue excised from patients with uterine leiomyoma. CD44-positive mesenchymal tumor stem-like cells were detected in both patients with intravenous leiomyomatosis and uterine leiomyosarcoma. The oncological properties of intravenous leiomyomatosis were found to be similar to those of uterine leiomyosarcoma. However, in intravenous leiomyomatosis, cyclin E and Ki-67-positive cells were rare and no pathological findings suspecting malignancy were observed. It is expected that establishing a treatment method targeting cancer stem cells will lead to the treatment of malignant tumors with a low risk of recurrence and metastasis.

## 1. Introduction

Uterine leiomyoma, also known as fibroids, is the most common benign neoplasm of the female genital tract. The prevalence of uterine leiomyoma in adult women over 50 is approximately 70%; therefore, uterine leiomyomas usually affect women in their fifties [1]. Uterine leiomyomas are discrete, round, firm, and often contain multiple uterine tumors composed of smooth muscle and connective tissue. Leiomyoma is the most common uterine tumor, and its subtypes account for approximately 10% of leiomyomas [1]. Leiomyomas are most prevalent among African-American women and least common among Asian women. Intravenous leiomyomatosis is an intravascular growth of benign smooth muscle cells in the absence of, or beyond the confines of, a leiomyoma, sometimes with pelvic or extrapelvic extension. Intravenous leiomyomatosis is more commonly observed in the uterus and rarely involves the broad ligament, pelvic veins, and vena cava [2]. Patients with intravenous leiomyomatosis have symptoms similar to those encountered in those with leiomyomas; less commonly, they present with chest pain, dyspnea, syncope, or pulmonary embolism due to right heart or pulmonary artery involvement. Pelvic magnetic resonance imaging may help detect early-stage disease, whereas computed tomography (CT) angiography and contrast-enhanced CT are useful in cases with extensions to extrapelvic vasculature [2].

Among patients with intravenous leiomyomatosis, approximately 30% have extrauterine extension involving the pelvic veins, inferior vena cava, and, rarely, heart or pulmonary vessels, leading to sudden death [3]. Recurrence, the risk of which is approximately 10%, may occur years later, either within the veins or rarely as benign metastasizing leiomyoma. Metastasizing leiomyoma is an extrauterine (most commonly in the lung), well-demarcated, and often a nodular proliferation of benign-appearing smooth muscle in patients with a history of uterine leiomyoma(s) [4]. Metastasizing leiomyoma lesions represent the spread from a histologically benign uterine smooth muscle tumor. Typically, uterine leiomyomas are benign tumors that do not invade into the vein. Therefore, a unique intracellular factor might be expressed in intravenous leiomyomatosis, which explains the peculiar infiltration into blood vessels that uterine leiomyoma does not have [1,2].

Histopathological studies showed intravascular growth of benign smooth muscle cells resembling typical leiomyoma or its subtypes in the absence of, or outside, a leiomyoma [2]. Moreover, hydropic change, hyalinization, and thick-walled vessels are frequent. Rarely, endometrial stroma and glands may be admixed with the smooth muscle component (termed as intravascular adenomyomatosis).

In uterine leiomyosarcoma, a malignant tumor, lymphatic metastasis is rare (frequency of ≤10%), whereas hematogenous metastasis is common [5,6]. There may be similarities between the physiological actions of the unique intracellular factors of intravenous leiomyomatosis and the hematogenous metastatic potential of uterine leiomyosarcoma. A small population of stem-like malignant tumor cells (i.e., malignant tumor stem cells) migrates to distant organs via intravascular infiltration and the construction of micrometastases. Previous studies have reported that clusters of differentiation (CD)13, CD44, and CD133 are cancer stem cell markers [7]. Mesenchymal stem cells (MSCs) are pluripotent cells with self-renewal capabilities found in the stroma of nonhematopoietic bone marrow. The known molecular markers expressed in MSCs include CD105 (SH2), CD73 (SH3/4), CD44, CD90 (Thy-1), CD71, and Stro-1, as well as the adhesion molecules CD106, CD166, and CD29 [8,9]. A comprehensive examination of these reports suggests that CD44 is an appropriate marker for uterine mesenchymal tumor stem cells.

Therefore, we examined the pathological features, including the population of tumor stem-like cells, in intravenous leiomyomatosis and uterine leiomyosarcoma using molecular pathological studies. The molecular pathological features common to intravenous leiomyomatosis and uterine leiomyosarcoma were observed. Similar to uterine leiomyosarcoma, many mesenchymal tumor stem-like cells, such as CD44-positive mesenchymal tumor cells, believed to have the ability to infiltrate into the vasculature, were found in intravenous leiomyomatosis tissue. The results obtained from this molecular pathological analysis contribute to developing inhibitors of hematogenous metastasis in intravenous leiomyomatosis, metastasizing leiomyoma, and uterine leiomyosarcoma.

## 2. Materials and Methods

### 2.1. Immunohistochemistry (IHC)

IHC staining for caveolin 1, cyclin B, cyclin E1, large multifunctional peptidase 2/b1i (LMP2/b1i), and Ki-67 was performed on serial human uterine mesenchymal tumor sections obtained from patients with uterine mesenchymal tumor (Supplementary Material 1). The monoclonal antibody for cyclin E1 (CCNE1/2460) was purchased from Abcam (Cambridge Biomedical Campus, Cambridge, UK), and the monoclonal antibody for Ki-67 (clone MIB-1) was purchased from Dako Denmark A/S (DK-2600 Glostrup, Denmark). The monoclonal antibody for caveolin 1, the monoclonal antibody for cyclin B1, and the monoclonal antibody for LMP2/b1i were purchased from Santa Cruz Biotechnology Inc. (Santa Cruz, CA, USA). The monoclonal antibody for CD44 was purchased from R&D Systems, Inc. (Minneapolis, MN, USA). IHC was performed using the avidin–biotin complex method, as described previously [10,11]. Briefly, one representative 5 mm tissue section was cut from a paraffin-embedded sample of a radical hysterectomy specimen from each patient with a uterine mesenchymal tumor.

Next, the sections were incubated with a biotinylated secondary antibody (Dako, DK-2600 Glostrup, Denmark) and then incubated with a streptavidin complex (Dako). The completed reaction was developed by 3, 39-diaminobenzidine, and the slide was counterstained with hematoxylin. Normal myometrium portions in the specimens were positive controls. The negative controls comprised tissue sections incubated with normal rabbit IgG instead of the primary antibody. Shinshu University approved these experiments according to internal guidelines (Approval No. M192). The expression of cyclin E and Ki-67 is indicated by brown 3,3’-diaminobenzidine tetrahydrochloride staining. Normal rabbit or mouse antiserum was a negative control for the primary antibody. The entire brown 3,3’-diaminobenzidine tetrahydrochloride-stained tissue was scanned with a digital microscope BZ-X800 (Keyence, Osaka, Osaka, Japan). Black dots indicate the expression of cyclin E and Ki-67.

### 2.2. Ethical Approval and Consent to Participate

This study was reviewed and approved by the Central Ethics Review Board of the National Hospital Organization Headquarters in Japan (Tokyo, Japan) and Shinshu University (Nagano, Japan). The exact date when the ethical approval was obtained was 17 August 2019. The code number of the ethical approval was NHO H31-02. The authors attended educational lectures on medical ethics in 2020 and 2021, which were supervised by the Japanese government. The completion numbers for the authors are AP0000151756, AP0000151757, AP0000151769, and AP000351128. Consent to participate was required, as this research was a clinical research. Subjects signed the informed consent when they were briefed on the clinical study and agreed with the contents of the clinical research. The authors attended a seminar on the ethics of experimental research using small animals on 2 July 2020, and 20 July 2021. They became familiar with the importance and ethics of animal experiments (National Hospital Organization Kyoto Medical Center and Shinshu University School of Medicine). The code number of the ethical approval for experiments with small animals was KMC R02-0702.

### 2.3. CD44-Positive Cell Selection

We purchased the established human uterine leiomyosarcoma (Ut-LMS) primary cell line, SK-LMS-1 (ATCC^®^ HTB-88^™^), from the American Type Culture Collection (ATCC) (Manassas, VA, USA). After cell selection, sterile cells were required, and the entire procedure was performed in a laminar flow hood to maintain sterile conditions. We isolated a candidate population of CD44-positive SK-LMS-1 subclone as human Ut-LMS stem-like cells from human Ut-LMS primary cells and SK-LMS-1 cells, using the CD44-positive selection method with MagCellect Plus Human CD44+ Cell Isolation Kit (R&D Systems, Inc., Minneapolis, MN, USA).

SK-LMS-1 was suspended in cold 1× MagCellect^TM^ Plus Buffer at a cell density of 1 × 10⁷ cells/mL prior to beginning the procedure. Next, 1 × 10⁷ cells (1.0 mL volume) were transferred into a 15 mL conical centrifuge tube, and then, 25 μL of Human CD44 Biotinylated Antibody was added. The cell–antibody suspension was gently mixed, avoiding bubble formation, and incubated at 2–8 °C for 15 min. The cell suspension was washed after incubation by adding 9 mL of cold 1× MagCellect^TM^ Plus Buffer and centrifuged at 300× *g* for 8 min. The supernatant was removed, and the cell pellet was resuspended by gently pipetting 1 mL of cold 1× MagCellect^TM^ Plus Buffer into the tube.

Streptavidin Ferrofluid (50 μL) was added to the cell suspension, mixed gently, and incubated at 2–8 °C for 15 min. At the end of the incubation period, the cell suspension was washed by adding 9 mL of cold 1× MagCellect^TM^ Plus Buffer and centrifuged at 300× *g* for 8 min. The supernatant was removed, and the cell pellet was resuspended by gently pipetting 3 mL of cold 1× MagCellect^TM^ Plus Buffer into the tube. Next, the cell suspension was transferred to a 5 mL polystyrene round-bottom tube and placed in the MagCellect^TM^ Magnet for incubation for 8 min at room temperature. Magnetically tagged cells (CD44^+^ SK-LMS-1 subclone) migrated toward the magnet (the desired cells) and were recovered according to the manufacturer’s procedure. While the tube was in the magnet, a sterile transfer pipette was used to carefully aspirate the reaction suspension (CD44^−^ SK-LMS-1 subclone) before transferring it to another tube. Flow cytometry visualized isolated CD44+ cells. The selected cells were resuspended in 100 μL of 1× MagCellect^TM^ Plus Buffer and stained using 5 μL of Human CD44 AlexaFluor^®^ 647 Detection Antibody, followed by standard staining procedures.

### 2.4. Xenograft Studies for the Micrometastasis Model

Nude mice (BALB/cSlc-*nu/nu*, female, 7–8 weeks old; Japan SLC, Shizuoka, Japan) were injected subcutaneously with 1 × 10^7^ cells of the CD44^−^ SK-LMS-1 subclone (five clones) and CD44^+^ SK-LMS-1 subclone (five clones) with BD Matrigel Matrix (BD Biosciences, Franklin Lakes, NJ, USA) in 5 mg/mL of culture medium containing 15% fetal calf serum plus SmGM-2 SingleQuots (CAMBREX, East Rutherford, NJ, USA) at a volume of 100 μL. Nude mice (BALB/cSlc-*nu/nu*, female, 7–8 weeks old; Japan SLC) were also injected subcutaneously with 1 × 10^7^ cells of the HeLa-Scr.shRNA clones (five clones). Tumor formation was assessed every day, and 7 weeks after injection, the tumors were dissected for western blotting. Tumor volumes were calculated as (L × W × W)/2, where W represents the width, and L represents the length. Statistical analysis was performed on mean tumor volumes at the end of the study using Dunnett’s test.

Xenografted BALB/c *nu/nu* mice were sacrificed for molecular pathological studies 2 months after injection. Whole lung and primary tumor tissues were also harvested for hematoxylin and eosin staining. Harvested tumors in the primary sites and lungs after the indicated times were fixed and embedded in paraffin, sectioned, and subjected to hematoxylin and eosin staining following a standard procedure. The number of CD44^−^ SK-LMS-1 subclone (five clones) and CD44^+^ SK-LMS-1 subclone (five clones) tumor nodules in the alveolar tissues of mice was counted under a microscope (Nikon COOLSCOPE, Tokyo, Japan) 2 months after the injection. The experiments with BALB/c *nu/nu* mice were conducted at Shinshu University and the National Hospital Organization Kyoto Medical Center following institutional guidelines (Approval No. M192).

## 3. Result

Like malignant tumors, benign tumors are heterogeneous among patients, i.e., benign tumor tissue is a heterogeneous cell population containing many fibroblasts and tumor stem cells other than tumor cells. Hematogenous metastases are present in many patients with uterine leiomyosarcoma. Recently, we treated a patient with intravenous leiomyomatosis (Supplementary Material 2). Even in intravenous leiomyomatosis, we noticed increased tumor growth/burden in the vein. Tumor stem cells are involved in tumor relapse, dissemination to distant organs, and resistance to antitumor agents [12]. Therefore, understanding the oncological properties of intravenous leiomyomatosis might contribute to developing new targeted antitumor agents for malignant mesenchymal tumors such as uterine leiomyosarcoma.

Proteasome is a proteolytic enzyme complex consisting of multiple subunits that degrades ubiquitinated proteins in eukaryotic cells and plays a central role in proteolytic degradation. Stimulation of IFN-γ induces the expression of beta subunits and constitutes the immunoproteasome, which regulates gene expression and cell proliferation by controlling the degradation of intracellular proteins. The expression of the major histocompatibility complex-linked low-molecular-mass polypeptide 2/β1i (LMP2/β1) subunit, which is increased by treatment with IFN-γ, amplifies specific endopeptidase activities of the immunoproteasome. Reports have demonstrated that uterine mesenchymal malignant tumors, i.e., uterine leiomyosarcoma (uLMS), spontaneously develop in *Lmp2/β1i*-deficient female mice at 6 months of age [13,14,15]. Studies have also shown that the prevalence of uLMS in *Lmp2/β1i*-deficient mice is approximately 37% at 12 months of age [13,14,15]. Therefore, clinical research performed by a collaboration of medical institutions examined the expression status of LMP2/β1i in 74 cases with normal myometrium, uterine leiomyoma, uLMS, and other uterine mesenchymal tumor tissues obtained from pathological files by immunohistochemical (IHC) staining using an antihuman LMP2/β1i monoclonal antibody [16,17]. Hematogenous metastases were also found in *Lmp2/β1i*-deficient female mice [13,14,15]. The incidence of other malignancies, including hepatocellular carcinoma, in *Lmp2/β1i*-deficient mice has been reported to be ≤1% [13,14,15]. Moreover, recent human clinical research has shown that the LMP2/β1i expression level was significantly lower in uLMS tissues than uterine leiomyoma and normal myometrium tissues.

Based on the markedly reduced expression of LMP2/β1i, candidate biomarkers specifically expressed in uLMS have been sought using genome-wide experimental methods with human tissues. As a result, caveolin 1, cyclin B, cyclin E, Ki-67/MIB1, and LMP2/β1i were identified as biomarker candidate factors specifically expressed in uLMS. A differential diagnostic method with IHC staining using a combination of several monoclonal antibodies against LMP2/β1i and other candidate cellular factors, such as caveolin 1, cyclin B, cyclin E, Ki-67/MIB1, and CD44, has been investigated for uterine mesenchymal tumors, including uLMS (Table 1) [18,19].

**Figure 1:**
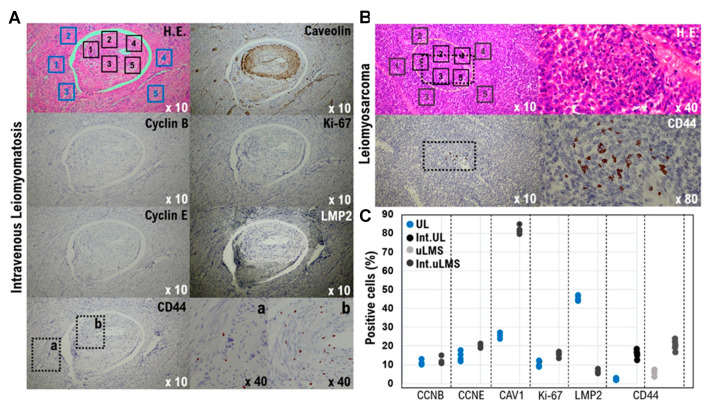
Differential expressions of factors cyclin B, cyclin E, caveolin 1, Ki-67, LMP2/b1i, and CD44 as potential biomarkers in intravenous leiomyomatosis and intravenous leiomyosarcoma. (**A**) Extensive intravascular growth of focal cellular smooth muscle is noted, forming worm-like plugs within veins. Focal hydropic change and components of endometrial glands are also observed. CD44-positive cells in the external tissue of intravenous are shown in the box **a**. CD44-positive cells in the internal tissue of intravenous are shown in the box **b**. (**B**) Intravenous leiomyosarcoma is shown in the upper right photograph. In uterine leiomyosarcoma, spindle-shaped tumor cells with round nuclei proliferate solidly. Compared with leiomyoma, uterine leiomyosarcoma shows increased nuclear density, nuclear hypertrophy, nuclear irregularities, and increased fission. (**C**) Immunohistochemistry of intravenous leiomyomatosis and intravenous leiomyosarcoma tissue sections was performed using appropriate monoclonal antibodies with standard procedures. The five tissue sites were randomly selected from intravenous leiomyomatosis’s internal and external tissues (i.e., normal uterine leiomyoma). As with uterine leiomyoma, the five tissue sites were randomly selected from the internal tissue of intravenous leiomyosarcoma. In a 40× field of view, the positive rates of the six factors were calculated in the five tissue sites. The positive rates at the sites of each tissue are shown in the scatter plot. Int.UL: Intravenous leiomyomatosis, Int.uLMS: Intravenous uterine leiomyosarcoma.

The expression status of five biomarker candidate factors and CD44 was examined by molecular pathological analysis using IHC to understand the biological characteristics of intravenous leiomyomatosis obtained from a patient with uterine leiomyoma (Supplementary Material 2). As a specific analysis method, five tissue sites were randomly selected from the internal and external tissues of intravenous leiomyomatosis (i.e., normal uterine leiomyoma) (Figure 1). The expression status of five biomarker candidate factors and CD44 was examined at each tissue site. Additionally, five tissue sites were randomly selected from the internal and external tissues of intravenous uterine leiomyosarcoma (i.e., normal uterine leiomyosarcoma) (Figure 1). The expression status of CD44 was examined at each tissue site of uterine leiomyosarcoma. Expressions of cyclin B, cyclin E, and Ki-67 were observed in the tissues of many cases of uterine leiomyoma. Molecular pathological analysis using IHC demonstrated that the positive rates of cyclin B, cyclin E, and Ki-67 in the internal tissue of intravenous leiomyomatosis were slightly higher than those of normal uterine leiomyoma (i.e., external tissue of intravenous leiomyomatosis) (Figure 1). Moreover, the positive rate of caveolin 1 in the internal tissues of intravenous leiomyomatosis was high compared with that in normal uterine leiomyoma (Figure 1, Table 1). However, in normal uterine leiomyoma, a high positive rate of LMP2/b1i was observed, but the expression of LMP2/b1i in the internal tissue of intravenous leiomyomatosis was negative (Figure 1, Table 1). Negative expression of LMP2/b1i in intravenous leiomyomatosis resembles previously reported biological features of uterine leiomyosarcoma. In uterine leiomyosarcoma, expression of CD44 was observed, but the expression of CD44 was not observed in normal uterine leiomyoma (Figure 1, Table 1). Many CD44-positive cells (i.e., mesenchymal tumor stem-like cells) are found in the internal tissue of intravenous leiomyomatosis, as well as in the internal tissue of intravenous leiomyosarcoma (Figure 1, Table 1). Mesenchymal tumor stem-like cells can invade the vasculature.

**Figure 2:**
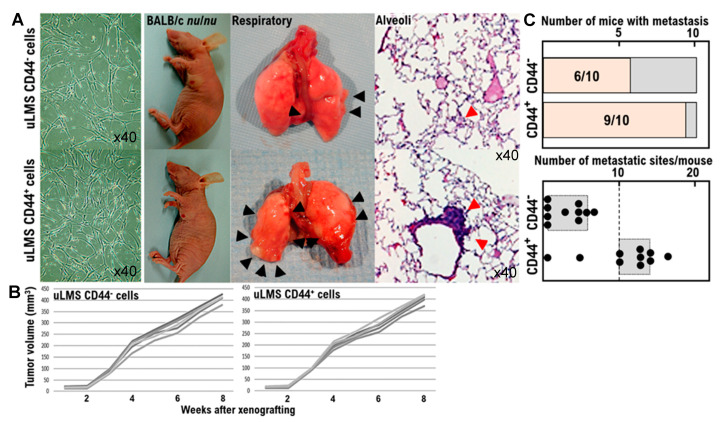
Induction of micrometastases in alveolar tissues derived from BALB/c *nu/nu* mice xenografted with human CD44-positive SK-LMS-1 stem-like cells. (**A**) Photographs show xenografts derived from BALB/c *nu/nu* mice inoculated with CD44-negative SK-LMS-1 cells or CD44-positive SK-LMS-1 cells at 2 months after the injection. Xenografts derived from BALB/c *nu/nu* mice inoculated with CD44-positive SK-LMS-1 cells were highly angiogenic. Data are representative of one of ten independent experiments obtained with different xenograft samples derived from CD44-negative SK-LMS-1 cells or CD44-positive SK-LMS-1 cells. The photographs show the pathology in metastasizes of respiratory tissue derived from the primary tumor on the left side of the second mammary fat pad, and micrometastases in the alveolar tissues of BALB/c *nu/nu* mice xenografted with CD44-negative SK-LMS-1 cells or CD44-positive SK-LMS-1 cells at 2 months after injection. In xenograft experiments using BALB/c *nu/nu* mice, the tumorigenicity of CD44-negative SK-LMS-1 cell and CD44-positive SK-LMS-1 cell xenografts was similar. Data are representative of one of ten independent experiments obtained with different xenograft samples derived from CD44-negative SK-LMS-1 cells or CD44-positive SK-LMS-1 cells. Metastatic tissues are indicated by black arrows. Micrometastasis in the alveoli are indicated by red arrows. (**B**) CD44-negative SK-LMS-1 cells or CD44-positive SK-LMS-1 cells were harvested from the cell culture using trypsin, washed, and resuspended in PBS (2 × 10^7^ cells/mL). Nude mice (BALB/cSlc-*nu/nu*, female, 7–8 weeks old; Japan SLC, Shizuoka, Japan) were injected with 1 × 10^6^ CD44-negative SK-LMS-1 cells or CD44-positive SK-LMS-1 cells in 5 mg/mL BD Matrigel Matrix (BD Biosciences) in culture medium containing 15% fetal calf serum plus SmGM-2 SingleQuots (Lonza, Basel) at a volume of 100 μL on the left side of the second mammary fat pad. Tumor formation was assessed every day. Tumor volumes were calculated as (L × W × W)/2, where W represents the width, and L represents the length. Statistical analyses were performed on mean tumor volumes at the end of the study using Student’s *t*-test. (**C**) The upper graph shows the number of mice in which micrometastases were observed in alveoli. The lower graph shows the number of micrometastatic sites found in alveolar sections in each xenografted mouse. It is important to note that in CD44-positive SK-LMS-1 cell-xenografted mice, the frequency of micrometastases in alveolar tissue was significantly higher than that in CD44-negative SK-LMS-1 cell-xenografted mice. Compared with CD44-negative SK-LMS-1 cells, CD44-positive SK-LMS-1 cells exhibit the ability to induce tumor angiogenesis and have higher hematogenous metastatic potential.

To examine the oncological properties of CD44-positive cells (i.e., uterine mesenchymal tumor stem-like cells), we isolated a candidate population of CD44-positive SK-LMS-1 subclone as human Ut-LMS stem-like cells from human Ut-LMS primary cells, SK-LMS-1 cells using the CD44-positive selection method with the MagCellect Plus Human CD44+ Cell Isolation Kit [20,21]. Tumor growth was clearly observed in control BALB/c *nu/nu* mice inoculated with the CD44^−^ SK-LMS-1 subclone (normal human LMS subclone cell) fraction; however, no reduction in tumor growth was observed in BALB/c *nu/nu* mice inoculated with the CD44^+^ SK-LMS-1 subclone (human LMS stem-like cells) (Figure 2, Supplementary Material Table S1). Moreover, no significant differences were observed in xenograft growth between the CD44^-^ SK-LMS-1 subclone and the CD44^+^ SK-LMS-1 subclone (Figure 2, Supplementary Mterial Table S1). Xenografts derived from BALB/c *nu/nu* mice inoculated with the CD44^+^ SK-LMS-1 subclone demonstrated higher angiogenic malignancy than those derived from BALB/c *nu/nu* mice inoculated with the CD44^−^ SK-LMS-1 subclone (Figure 2). It is important to note that the number of micrometastases in alveolar tissues was significantly higher in BALB/c *nu/nu* mice xenografted with the CD44^+^ SK-LMS-1 subclone than in BALB/c *nu/nu* mice xenografted with the CD44^−^ SK-LMS-1 subclone (Figure 2, Supplementary Material Table S1). Compared with the xenograft model of CD44-negative cells, there were more micrometastases in the alveoli in the xenograft model of CD44-positive cells (i.e., mesenchymal tumor stem-like cells) (Figure 2, Supplementary Material Table S1). This study showed that CD44-positive cells have hematogenous metastatic potential associated with vascular infiltration (Figure 2, Supplementary Material Table S1). However, these findings do not provide medical evidence that CD44-positive cells have stronger tumor growth potential compared with the xenograft model of CD44-negative cells. VEGF-A secreted from the primary tumors of BALB/c *nu/nu* mice xenografted with the CD44^+^ SK-LMS-1 subclone may promote hematogenous metastasis. Further studies should examine the intravascular transferability of CD44-positive mesenchymal tumor cells isolated from tissues of intravenous leiomyomatosis (i.e., uterine mesenchymal tumor stem-like cells).

**Figure 3:**
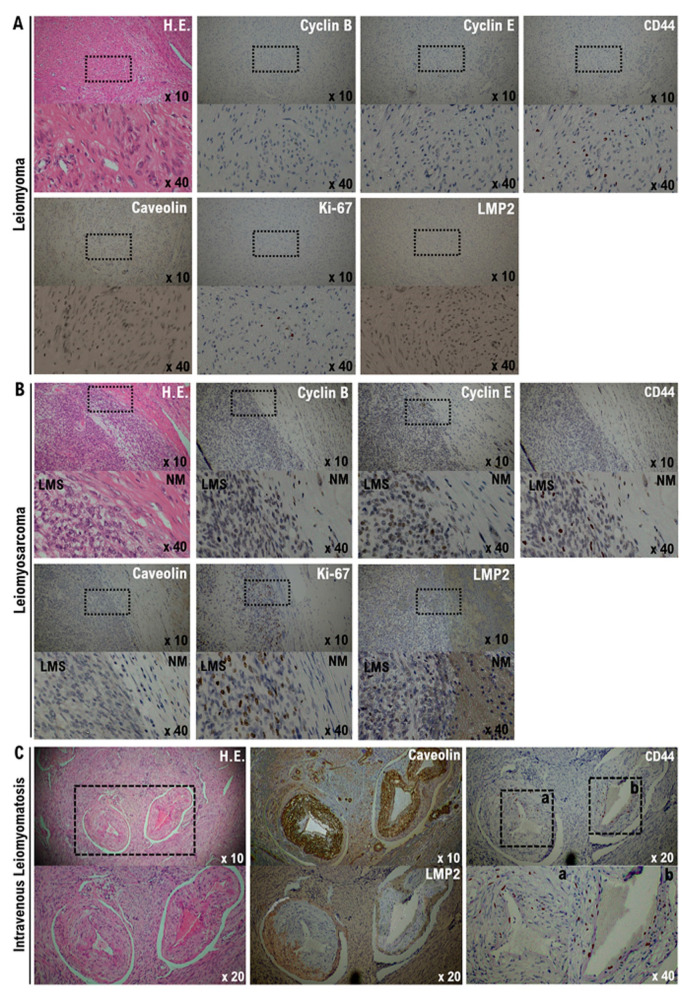
Differential expression of cyclin B, cyclin E, caveolin 1, Ki-67, LMP2/b1i, and CD44 as potential biomarkers in leiomyoma, leiomyosarcoma, and intravenous leiomyomatosis. (**A**) The photograph shows spindle cell leiomyoma. The low power view (10× field) shows a well-circumscribed tumor nodule in the myometrium composed of broad fascicles of spindle cells. High power view (40× field) shows that uterine leiomyoma (spindle cell) has bland cytological features, with elongated nuclei and fine nuclear chromatin. Immunohistochemistry of leiomyoma tissue was performed using appropriate monoclonal antibodies with standard procedures. (**B**) The photograph shows epithelioid leiomyosarcoma. The low power view (10× field) shows the uterine mass irregular interface with the myometrium, composed of round to polygonal cells with granular eosinophilic cytoplasms. Notice the presence of significant nuclear atypia and clear mitoses. The high power view (40× field) shows that the tumor cells are round to ovoid, with eosinophilic granular cytoplasm and irregularly shaped nuclei. Immunohistochemistry of leiomyoma tissue was performed using appropriate monoclonal antibodies with standard procedures. (**C**) Intravenous leiomyomatosis is a rare neoplasm characterized by nodular masses of histologically benign smooth muscle cells growing within the venous system. Significant smooth muscle hyperplasia was observed in the myometrium. Some tumors invade venous blood vessels. The low power view (10× field) shows no obvious high-grade nuclear atypia or mitotic cell proliferation and necrosis. The high power view (40× field) shows interlaced bundles of homogeneous spindle cells with oval nuclei, eosinophilic cytoplasm, rare mitotic figures, and decorated with several thick-walled small blood vessels, which are consistent with features of leiomyoma. Immunohistochemistry of leiomyoma tissue was performed using appropriate monoclonal antibodies with standard procedures. The areas surrounded by the dotted squares are enlarged.

**Figure 4:**
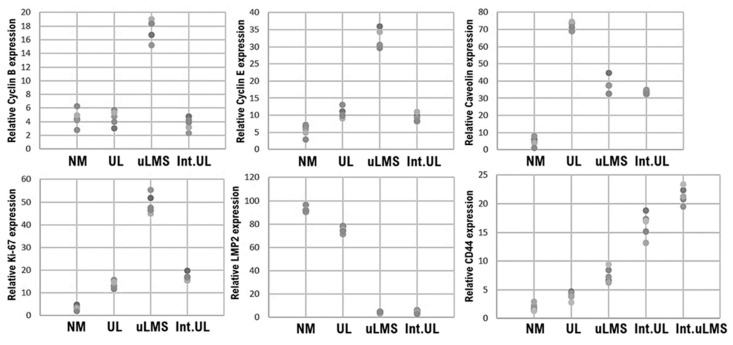
CD44-positive uterine mesenchymal tumor stem-like cells in intravenous leiomyomatosis and intravenous uterine leiomyosarcoma. Immunohistochemistry of normal myometrium, uterine leiomyoma, uterine leiomyosarcoma, intravenous leiomyomatosis, and intravenous leiomyosarcoma tissues was performed using appropriate monoclonal antibodies with standard procedures. The five tissue sites were randomly selected: normal myometrium, uterine leiomyoma, uterine leiomyosarcoma, internal tissue of intravenous leiomyomatosis, and intravenous leiomyosarcoma. In a 40× field of view, the positive rates of the six factors were calculated in the five tissue sites under microscopy (Panthera Shimadzu Co. Ltd., Kyoto, Japan); the positive rates at the sites of each tissue are shown in the scatter plot. Int.UL: Intravenous leiomyomatosis, Int.uLMS: Intravenous uterine leiomyosarcoma.

In clinical studies to date, the differential expression status of five factors (caveolin 1, cyclin B, cyclin E, Ki-67/MIB1, and LMP2/β1i) in the tissues of various uterus mesenchymal tumors of (normal mesenchymal, uterine leiomyoma, leiomyoma with Bizarre Nuclei, STUMP, leiomyosarcoma, LANT) has been reported [22]. Therefore, we examined the expression status of five factors and CD44 in the tissues of intravenous leiomyomatosis and compared them with the expression status of each factor in various mesenchymal tumors. IHC analysis demonstrated the expression of cyclin B, cyclin E, and Ki-67 in the tissue of uterine leiomyoma, albeit with lower positive rates (Figure 3A). In contrast, in uterine leiomyoma, caveolin and LMP2/b1i were strongly expressed throughout the tissue, and the positive rate of these three factors was high (Figure 3A and Figure 4). Next, the molecular pathological analysis revealed the expression of cyclin B, cyclin E, and Ki-67 in the tissues of uterine leiomyosarcoma, with high positive rates (Figure 3B). However, the expression of cyclin B, cyclin E, and Ki-67 was unclear in normal myometrium tissue (Figure 3B and Figure 4, Supplementary Material Table S2). In addition, in uterine leiomyosarcoma tissue, caveolin was strongly expressed throughout the tissue (Figure 3B and Figure 4, Table 1, Supplementary Material Table S2).

Although some T lymphocytes express CD44, it is possible that the CD44-positive cells are not T lymphocytes. Recent reports demonstrated that uterine mesenchymal tumors including uterine leiomyosarcomas were mismatch repair-deficient [23,24,25]. Uterine leiomyosarcoma, 1.4% of stage I–III malignant tumors and 0.6% of stage IV malignant tumors, were classified in dMMR-positive tumors [23,24,25]. From these research findings, in uterine leiomyosarcoma, the numbers of infiltrating CD8-positive T cells are considered to be small. Therefore, as previously reported, the antitumor effect of therapy by immune checkpoint inhibitors on uterine leiomyosarcoma is considered to be low [23,24,25].

In contrast, LMP2/b1i was strongly expressed throughout the tissue in normal myometrium, but in uterine leiomyosarcoma tissue, LMP2/β1i expression was not observed (Figure 3B and Figure 4, Table 1, Supplementary Material 4). As shown in Figure 1, CD44-expressing cells were found in uterine leiomyosarcoma. Therefore, we examined the presence of CD44-positive cells in the internal tissue of intravenous leiomyomatosis using the excised tissue obtained from other patients with uterine leiomyoma. In the normal uterine leiomyoma, the expression of CD44 was not clearly observed (Figure 3C and Figure 4, Table 1). Many CD44-positive cells were found in the internal tissue of intravenous leiomyomatosis and the internal tissue of intravenous uterine leiomyosarcoma (Figure 3C and Figure 4, Table 1).

## 4. Discussion

One in four women is affected by uterine leiomyomas-benign tumors of the uterine wall, also known as uterine fibroids, during their premenopausal life. Uterine leiomyomas can cause excessive bleeding, pain, and infertility [26] and are a common cause of hysterectomy [27]. Uterine leiomyomas reportedly emerge through at least three distinct genetic drivers: mutations in the mediator complex subunit 12 or fumarate hydratase or genomic rearrangement of high mobility group at-hook 2 [28]. Moreover, uLMS have been shown to spontaneously develop in *Lmp2/β1i*-deficient female mice after 6 months of age [13,14,15]. Studies have shown that the prevalence of uLMS in *Lmp2/β1i*-deficient mice is approximately 37% at 12 months of age [13,14,15].

To identify pathological variants of uterine leiomyosarcoma based on the results obtained from studies using *Lmp2/β1i*-deficient mice, we conducted a clinical study using excised tissues obtained from patients with uterine mesenchymal tumors, including uterine leiomyosarcoma. Similar to *Lmp2/β1i*-deficient mice, our results demonstrated defective expression of an LMP2/β1i transcript in uterine leiomyosarcoma. This finding suggests that defective expression of the LMP2/β1i transcript is directly involved in the development of uterine leiomyosarcoma. Similarly, in intravenous leiomyomatosis, defective expression of the LMP2/β1i transcript was also observed. In uterine leiomyosarcoma, distant metastasis due to intravascular infiltration of tumor cells, which is frequently observed, and intravascular infiltration of tumor cells in intravenous leiomyomatosis are considered to be controlled by the same key factor. Presumably, the signal cascade induced by impaired expression of LMP2/β1i is associated with the intravascular infiltration ability of tumor cells.

The oncological properties of intravenous leiomyomatosis are similar to those of uterine leiomyosarcoma. However, intravenous leiomyomatosis is a benign tumor that differs significantly from uterine leiomyosarcoma, with a 5-year survival rate of <20%. Recent reports have demonstrated that cyclin E-deficient cells actively proliferate in conditions of continuous cell cycling but are unable to re-enter the cell cycle from the G_1_ phase to the S phase and are resistant to chemical-induced oncogenic transformation [29,30,31,32]. Cyclin E, a regulator of the cell cycle, and Ki-67/MIB1, a diagnostic biomarker in proliferating cancer or malignant tumor cells, affect the behavior of human breast cancer cells and uLMS [32,33,34]. Clinical studies have suggested that patients with uLMS with high expression levels of cyclin E and Ki-67/MIB1 have a poor prognosis. Therefore, the expression status of cyclin E and Ki-67/MIB1 correlates with the malignant potential of uterine mesenchymal tumors.

Intravenous leiomyomatosis is an intravascular growth of benign smooth muscle cells in the absence of or beyond the confines of a leiomyoma, sometimes with pelvic or extrapelvic extension. Therefore, the oncological properties of growth of intravenous leiomyomatosis are similar to the oncological characteristics of uterine leiomyosarcoma. However, no metastatic lesions are found in distant organs such as the lung and liver in patients with intravenous leiomyoma. In our clinical research with a large cohort, poor-prognosis uterine mesenchymal tumors, i.e., uterine leiomyosarcoma, have high positive rates for cyclin E and Ki-67. Therefore, we believe that the high positive rates of cyclin E and Ki-67 are prognostic markers for uterine mesenchymal tumors. In intravenous leiomyomatosis, cyclin E and Ki-67-expressing cells, which may be associated with prognosis, were not observed in our study. Therefore, unlike uterine leiomyosarcoma, intravenous leiomyomatosis is considered to have different properties from malignant tumors with a poor prognosis.

Cancer stem cells play an important role in the formation and growth of cancer and are associated with metastasis and recurrence. Previous studies have indicated that CD13, CD44, and CD133 are markers for cancer stem cells. Moreover, in MSCs, CD105 (SH2), CD73, CD44, CD90, CD71, and Stro-1 are known molecular biomarkers, as are the adhesion molecules CD106, CD166, and CD29. A comprehensive examination of these reports suggests that CD44 is an appropriate marker for uterine mesenchymal tumor stem cells.

**Figure 5:**
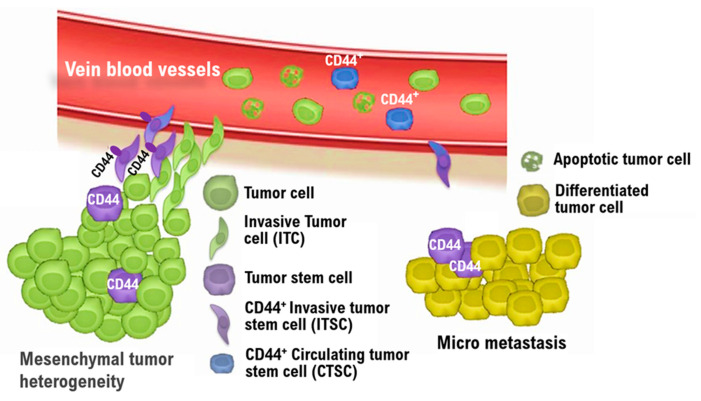
The invasion and metastatic process of uterine mesenchymal tumor stem-like cells. The nature of malignant tumors varies from patient to patient. Malignant tumor tissue comprises a heterogeneous cell population, containing many fibroblasts and tumor stem cells in addition to tumor cells. The heterogeneity of malignant tumor cells is one of the main reasons for the difficulty in treating malignant tumors. In particular, malignant tumor stem cells undergo distant metastasis and have resistance to antitumor agents. Understanding the oncological properties of malignant tumor stem cells contributes to the development of new targeted antitumor agents. The complex process of distant metastasis includes detachment, invasion of the tumor microenvironment, and shedding of invasive tumor cells (ITCs) and CD44-positive invasive tumor stem cells (ITSCs) into the bloodstream (intravasation). The majority of ITCs relate to differentiated cancer cells with low tumorigenicity and instead undergo apoptosis. However, CD44-positive ITSCs bear cancer stem cell features, i.e., CD44-positive circulating tumor stem cells (CTSC), survive in the circulating blood, escape from immune surveillance, and go home to secondary sites, extravasate, and ultimately form distant metastatic lesions. The application of cytotoxic drugs for ITSCs and/or CTSCs, such as celecoxib, greatly reduces the incidence of tumor metastases and tumor recurrence [35]. ITC: Invasive tumor cell, ITSC: Invasive tumor stem cell, CTSC: Circulating tumor stem cell.

Hematogenous metastases were reportedly also found in Lmp2/β1i-deficient female mice [13,14,15]. The incidence of other malignancies (i.e., hepatocellular carcinoma) in LMP2/β1i-deficient mice has been reported to be 1% or less [13,14,15]. We are now examining the physiological properties of uterine mesenchymal tumor stem-like cells in more detail. Similar to malignant tumors, benign tumors are heterogeneous among patients and contain many fibroblasts and tumor stem cells other than tumor cells. As intravenous leiomyomatosis is a disease in which tumors grow in veins, presumably, CD44-positive mesenchymal tumor stem-like cells have the capacity for intravascular infiltration. However, CD44-positive mesenchymal tumor stem-like cells may be sensitive to antitumor agents due to their low positive rates for cyclin E and Ki-67 (Figure 5). Understanding the oncological properties of intravenous leiomyomatosis might contribute to developing new targeted antitumor agents for malignant mesenchymal tumors such as uterine leiomyosarcoma.

## 5. Conclusions

Our work describes a potential mechanism of tumorigenesis and intravascular infiltration caused by uterine mesenchymal tumor stem-like cells. The effectiveness of chemotherapeutic, immunotherapy, and targeted agents on tumor cells, as well as the drug response, may be assessed according to tumor cell viability through released biomarkers from human uterine mesenchymal tumor stem-like cells. The characteristics of these human uterine mesenchymal tumor stem-like cells might provide insights into developing novel therapeutics and diagnostic methods for uterine mesenchymal tumors. Further oncological studies of mesenchymal tumors are required to establish treatments for poor-prognosis uterine leiomyosarcoma.

## Figures and Tables

**Table 1:** Differential expressions of PSMB9/β1i, CALPONIN h1 and CD44 in human uterine mesenchymal tumors and uterine LANT-like tumor.

	Age Years	*n*	LMP2/β1i Expression				CAVEOLIN 1 Expression				CD44		
			−	−/+ ^1^	Focal ^2^	+++	−	−/+ ^1^	Focal ^2^	+++	−	Focal ^2^	++
Normal	32–83	74				74	74				74		
Leiomyoma (Ordinary leiomyoma) (Cellular leiomyoma) (Tumour of uncertain malignant potential)	33–83	52 (30) (10) (12)				52				52	52		
Intravenous leiomyomatosis	48,51	2	2							2			2
Bizarre Leiomyoma	44–55	3				3				3			
Leiomyosarcoma	32–83	58	49	3	4	2	0	1	6	51			49
U.LANT-like tumor	45	1	1				1						

Staining score of LMP2/β1i expression, CALPONIN h1 expression and CD44 expression from results of IHC experiments. **−**; staining with less than 1.0% of cells stained, **−**/+ ^1^; partially positive (5% to 10% of cells stained), Focal ^2^; Focal-positive (focal or sporadic staining with less than 5% of cells stained), ++; staining with more than 5% and less than 90% of cells stained, +++; diffuse-positive (homogeneous distribution with more than 90% of cells stained), **−**; negative (no stained cells). U.LANT-like tumor; uterine leiomyomatoid angiomatous neuroendocrine tumor-like tumor.

## Data Availability

The data that support the findings of this study are available from the corresponding author, [author initials], upon reasonable request.

## Supplementary Materials

The following are available online at https://www.mdpi.com/article/10.3390/cimb43020084/s1.

## Abbreviations

CD	clusters of differentiation
CT	computed tomography
IHC	immunohistochemistry
LMP2/b1i	large multifunctional peptidase 2/b1i
LMS	leiomyosarcoma
MSC mesenchymal stem cell	mesenchymal stem cell
