# Supplementary material for: Oncological Properties of Intravenous Leiomyomatosis: Involvement of Mesenchymal Tumor Stem-Like Cells
Source: Curr Issues Mol Biol. 2021 Sep 19;43(2):1188–202. doi: 10.3390/cimb43020084 (PMC8929133; doi:10.3390/cimb43020084)
Supplement: Supplementary file 1 [file cimb-43-00084-s001.zip › cimb-1388610-supplementary.pdf]

Supplementary Materials

Oncological features of intravenous leiomyomatosis: involvement of mesenchymal tumor stem-like cells

Saya Tamura, Takuma Hayashi, Hideki Tokunaga, Nobuo Yaegashi, Kaoru Abiko, Ikuo Konishi

Supplementary material 1

Tissue Collection. A total of 101 patients between 32 and 83 years of age and diagnosed as having smooth muscle tumors of the uterus were selected from pathological files. Serial sections were cut from at least 2 tissue blocks from each patient for hematoxylin and eosin staining and immunostaining. All tissues were used with the approval of the Ethical Committee of Shinshu University after obtaining written consent from each patient. The pathological diagnosis of uterine smooth muscle tumors was performed using established criteria (Hendrickson and Kempson, 1995) with some modification. Briefly, usual leiomyoma (usual LMA) was defined as a tumor showing typical histological features with a mitotic index (MI) [obtained by counting the total number of mitotic figures (MFs) in 10 high-power fields (HPFs)] of <5 MFs per 10 HPFs. Cellular leiomyoma (cellular LMA) was defined as a tumor with significantly increased cellularity (>2000 myoma cells / HPF) and a MI<5, but without cytologic atypia. Bizarre leiomyoma (BL) was defined as a tumor either with diffuse nuclear atypia and a MI<2 or with focal nuclear atypia and a MI<5 without coagulative tumor cell necrosis. A tumor of uncertain malignant potential (UMP) was defined as tumor with no mild atypia and a MI<10 but with coagulative tumor cell necrosis. Leiomyosarcoma (LMS) was diagnosed in the presence of a MI>10 with either diffuse cytologic atypia, coagulative tumor cell necrosis, or both. Of the 105 smooth muscle tumors, 52 were diagnosed as LMA, 3 were BL, 2 were intravenous leiomyomatosis, 58 were uterine LMS and 1 was uterine LANT-like tumor. Of the 58 LMS, 48 were histologically of the spindle-cell type and 10 were of the epithelioid type. The clinical stage of the LMS patients was stage I in 11 cases, stage II or III in 31 cases, and stage IV in 16 cases. Protein expression studies with cervix epithelium and carcinoma tissues were performed using tissue array (Uterus cancer tissues, AccuMax Array, Seoul, Korea). Details about tissue sections are indicated in manufacture's information (AccuMax Array).

Supplementary material 2

On May 10, 2020, a 45-year-old woman was alerted to the possibility of a chocolate cyst in the examination of the company. At another hospital, MRI showed a tumor in the pelvis, but its malignancy was unknown. However, because an enhancement component was observed in the masses, ovarian cancer was considered a possibility, and the patient was referred to our hospital. On October 29, 2020, at the first outpatient visit at our hospital, a slightly mobile right elastic soft mass was palpated. The bilateral parauterine tissues, i.e., the fallopian tubes and ovaries, were softened. Transvaginal ultrasonography revealed a mass in the posterior uterine muscularis in the right uterine

appendage area. The boundary between the right side of the uterus and the mass was unclear, as were the contours and boundaries of the mass. A mass measuring 115 × 57 mm in the retroperitoneal space was suspected as leiomyoma. However, it was also possible that this mass was a retroperitoneal tumor.

Contrast-enhanced abdominal computed tomography (CT) and MRI examinations showed that a mass lesion showing low to high signal on MRI T1WI had spread from the muscular layer of the right wall of the uterine body to the outside of the uterus. The mass was wrapped in a membrane and was located between the uterine circle ligament and the uterine artery. There was a normal right ovary on the cranial side of the mass. The diffusion-weighted image showed a slightly high signal and a high ADC map, but no mass diffusion was observed. From the results of these imaging tests, degenerative leiomyoma that grew from the myometrium into the broad ligament was considered. A leiomyoma of approximately 2 cm in size was also found on the posterior wall of the bottom of the uterus. There were no mass lesions in either ovary. Examination by contrast-enhanced abdominal CT revealed a liver cyst. No significant lymphadenopathy and ascites retention were observed. No obvious nodules were found in the lung field of view, and no pleural effusion was observed.

On March 21, 2021, a simple total hysterectomy and bilateral salpingectomy were performed. Laparoscopic findings included a small amount of pale, bloody ascites. Adhesions covering the bilateral sacral uterine ligaments appeared in the Douglas fossa due to the effects of endometriosis. A tumor of approximately 10 × 8 cm in size growing in the broad ligament was attached to the right circular ligament. Gross findings showed that the tumor had edematous hardness, suggesting degenerative uterine leiomyoma.

Surgical pathological diagnosis was performed on the excised uterine body and the excised bilateral fallopian tube tissue. Macroscopic findings showed a soft tumor approximately 13 cm in size protruding from the serosal surface of the uterus. The cut surface of the tumor was grayish-white and polynodular.

There was smooth muscle hyperplasia with island-like and alveolar-like morphology with edematous interstitium in the grayish-white nodules in the tumor. Significant smooth muscle hyperplasia was observed in the myometrium. Some tumors invaded venous blood vessels, but no obvious high-grade nuclear atypia or mitotic cell proliferation and necrosis were observed. Histological examination revealed that the resected tumor was cotyledonoid dissecting leiomyoma. However, normal leiomyoma was also observed. No malignant findings were found in the endometrium, cervix, or bilateral fallopian tubes.

Supplementary material Table 1

Xenografting model of human uterine mesenchymal tumor cells

Clone type	Tumor volume (mm^3)	micrometastasis	Number of metastatic sites/mouse
CD44$^-$ SK-LMS-1 #1	425.8	+	8
CD44$^-$ SK-LMS-1 #2	408.7	+	6
CD44$^-$ SK-LMS-1 #3	409.8	+	3
CD44$^-$ SK-LMS-1 #4	378.6	-	0
CD44$^-$ SK-LMS-1 #5	412.8	+	6
CD44$^-$ SK-LMS-1 #6	420.9	-	0
CD44$^-$ SK-LMS-1 #7	410.7	-	0
CD44$^-$ SK-LMS-1 #8	392.9	+	7
CD44$^-$ SK-LMS-1 #9	409.4	-	0
CD44$^-$ SK-LMS-1 #10	386.4	+	6
Average	405.6		3.6
CD44$^+$ SK-LMS-1 #1	408.7	+	14
CD44$^+$ SK-LMS-1 #2	398.2	+	13
CD44$^+$ SK-LMS-1 #3	369.3	-	0
CD44$^+$ SK-LMS-1 #4	416.7	+	14
CD44$^+$ SK-LMS-1 #5	420.2	+	10
CD44$^+$ SK-LMS-1 #6	379.2	+	6
CD44$^+$ SK-LMS-1 #7	403.9	+	13
CD44$^+$ SK-LMS-1 #8	410.8	+	10
CD44$^+$ SK-LMS-1 #9	398.5	+	13
CD44$^+$ SK-LMS-1 #10	401.7	+	17
Average	400.7		11

Tumor type	Atypia	Mitotic activity	Necrosis	Protein expression*													Cliical comments
				Cyt	Des	CAV	SMA	Vim	ER/PR	End	EGF	CyB	CyE	PSMB9	Cal	Ki67	
**Endometrial stromal tumors.**																	
Endometrial stromal nodule	minimal	infrequent	-/inconspicuous	+	-	++	+	+	+++	+	*	+	-	++	++	-	Absence of myometrial infiltration
Endometrial stromal sarcoma	-	infrequent	-/inconspicuous	-/+	-	++	+	+	+++	+	+	+	-	-/+	++	-/+	
Undifferentiated endometrial sarcoma	marked	Frequent (atypical MF)	+	-/+	foc	*	*	-	-	+	+	+	+	-/+	+	+	Lack specific differentiation
**Smooth muscle tumors**																	
Leiomyoma, NOS	-	<5 MF/10HPF	-	foc	+	++	+	*	+++	-/+	-/+	+	-	++	++	-/+	Well-circumscribed
Mitotically active leiomyoma	-	>5 MF/10HPF	-	*	+	++	+	*	+++	-/+	-/+	+	-	++	++	-/+	Pseudocapsul
Cellular leiomyoma	-	infrequent	-	*	+	++	+	*	+++	-/+	-/+	+	-	++	++	-/+	Increased cellularity
Hemorrhagic cellular leiomyoma	-	infrequent	-	*	+	++	+	*	+++	-/+	-/+	+	-	++	++	-/+	Hormone induced changes
Epithelioid leiomyoma	-	<5 MF/10HPF	-	*	+	++	+	*	+++	-/+	-/+	*	-	++	++	-/+	Epithelial-like cells
Myxoid leiomyoma	-	<5 MF/10HPF	-	*	*	*	*	*	*	-/+	-/+	*	+	-/+	+	+	Myxoid material
Atypical leiomyoma	moderate	<10 MF/10HPF	-	-	+	++	+	*	+++	+	-/+	+	+	-/+	-/+	+	Separates tumor cells
Lipoleiomyoma	-	infrequent	-	*	+	++	+	*	+++	*	-/+	*	+	-/+	*	-/+	Scattered adipocytes
STUMP#	-	infrequent	-	*	+	++	+	*	*	*	-/+	*	-/+	-/+	*	-/+	
	-/+	>10 MF/10HPF	+/uncertain	*	+	++	+	*	*	*	-/+	*	-/+	-/+	*	-/+	
	Marked	borderline	-	*	+	++	+	*	*	*	-/+	*	-/+	-/+	*	-/+	
	-	infrequent	+/difficult classify	*	+	++	+	*	*	*	-/+	*	-/+	-/+	*	-/+	
Intravenous leiomyomatosis	moderate	infrequent	-	+	*	++	+	*	*	*	*	-/+	-/+	-	-	+	Infiltrative
Leiomyosarcoma	moderate	>10 MF/10HPF	+	+	+	++	-/+	-	-	+	-/+	++	+++	-	-	++	Infiltrative
Leiomyosarcoma epitheliod variant	moderate	>5 MF/10HPF	+	+	+	++	-/+	-	-	+	-/+	++	+++	-	-	++	Infiltrative, >50% epithelioid cells
Leiomyosarcoma myxoid variant	moderate	Any MF	+	+	+	++	-/+	-	-	+	-/+	++	++	-	-	++	Infiltrative, myxoid extracellular matrix
**Leiomyomatoid tumor**																	
LANT#	absent	frequent	+	-	-	+	+	+	*	*	+	*	++	-	-	-/+	NOTE1

*insufficient data or not applicable.

Cyt.; cytokeratin, Des.; Desmin, CAV; caveolin 1, SMA; smooth muscle actin, Vim.; vimentin, ER/PR; estrogen receptor/progesterone receptor, End.; Endoglin; CD105/TGFb receptor (stem cell marker), EGF, EGFR; epidermal growth factor receptor, CyB; cyclin B1, CyE; cyclin E, PSMB9; proteasome beta subunit 9, Cal.; calponin h1, CD56; neural cell adhesion molecule (N-CAM), WT-1; wilms tumor 1, NOS; not otherwise specified, MF; magnification factor, HPF; high power field, Foc.; focal, STUMP; smooth muscle tumors of uncertain malignant potential. Protein expression*, estimated-protein expressions by immunoblot analysis, immunohistochemistry (IHC) and/or RT-PCR (quantitative-PCR), +/-; partial expression, +; expression, ++; medium expression, +++; high expression, -; no evidence of expression, ER/PR(ref.24), PSMB9 (ref.22,23), cyclin E(ref.24,32), calponin h1(ref.29,30,31), Ki-67(ref.24,33). STUMP#(ref.33,34). Cyclin E, PSMB9, calponin h1 are potential bio-marker for human uterine mesenchymal tumors. LANT#, leiomyomatoid angiomatous neuroendocrin tumour (LANT) is described as a dimorphic neurosecretory tumor with a leiomyomatous vascular component (ref.35,36). NOTE1, Low-grade neuroendocrine tumor possibly related to null cell adenoma.

Supplementary material Table 2. Classification of human uterine mesenchymal tumors
